# A Case of Torsion of the Gallbladder Suspected with SPECT-CT: Review and Recommendations

**DOI:** 10.1155/2020/8687141

**Published:** 2020-01-04

**Authors:** Eric Bergeron, Etienne Désilets, Xuan Vien Do, Daniel McNamara, Sami Chergui, Michael Bensoussan

**Affiliations:** Departments of Surgery, Gastroenterology and Medical Imaging, Charles-LeMoyne Hospital, Greenfield Park, Canada

## Abstract

Torsion or volvulus of the gallbladder is a rare situation that rapidly progresses to gangrene and linked with a poor prognosis, even death, if unrecognized and untreated. An interesting and rare case of gallbladder volvulus in which diagnosis was obtained by comparing CT images and HIDA scan with SPECT-CT is presented. Relevant literature is reviewed, and recommendations are outlined.

## 1. Introduction

Torsion or volvulus of the gallbladder, first described as early as 1898, is a rare entity that may be encountered only once in an entire practice [1]. Despite improvements in diagnostic imaging modalities, majority of the cases remain undiagnosed preoperatively [1]. Torsion of the gallbladder rapidly progresses to gangrene and is linked with a very poor prognosis, even death, if not recognized and treated in time [2–7].

We present an interesting case of gallbladder volvulus in which diagnosis was obtained by comparing CT images and HIDA scan with SPECT-CT. We reviewed the literature and propose some recommendations.

## 2. Case Presentation

An 84-year-old thin and frail woman (BMI = 17.3) with rotoscoliosis, severe osteoporosis, and vertebral fractures presented to the emergency room with back pain. Spine X-ray showed two new vertebral fractures at T7 and L1 as well as a possible ileus. She was afebrile and had no abdominal pain. She was discharged with analgesic.

**Figure 1:**
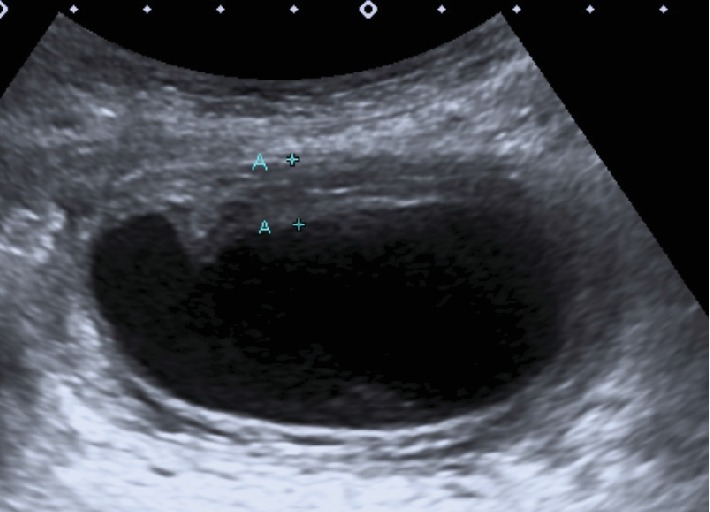
Gray-scale ultrasound depicting lamellated wall thickening (A+) of the gallbladder without cholelithiasis.

The following day, she presented to the emergency department because there was no relief of her back pain. She was now complaining of a right upper quadrant pain. Murphy's sign was negative. Temperature was 37.1°C, and heart rate was 100 bpm. White cell count was 19,000/mm^3^. Liver function parameters were within normal limits. An abdominal ultrasound showed distension of the gallbladder and lamellated wall thickening up to 9 mm, but no gallstones were seen (Figure 1). There was a small amount of free fluid around the liver. The patient was kept hospitalized. A 99mTc-labeled hepatobiliary iminodiacetic (HIDA) scintigraphy was ordered because of the possibility of an acute cholecystitis.

**Figure 2:**
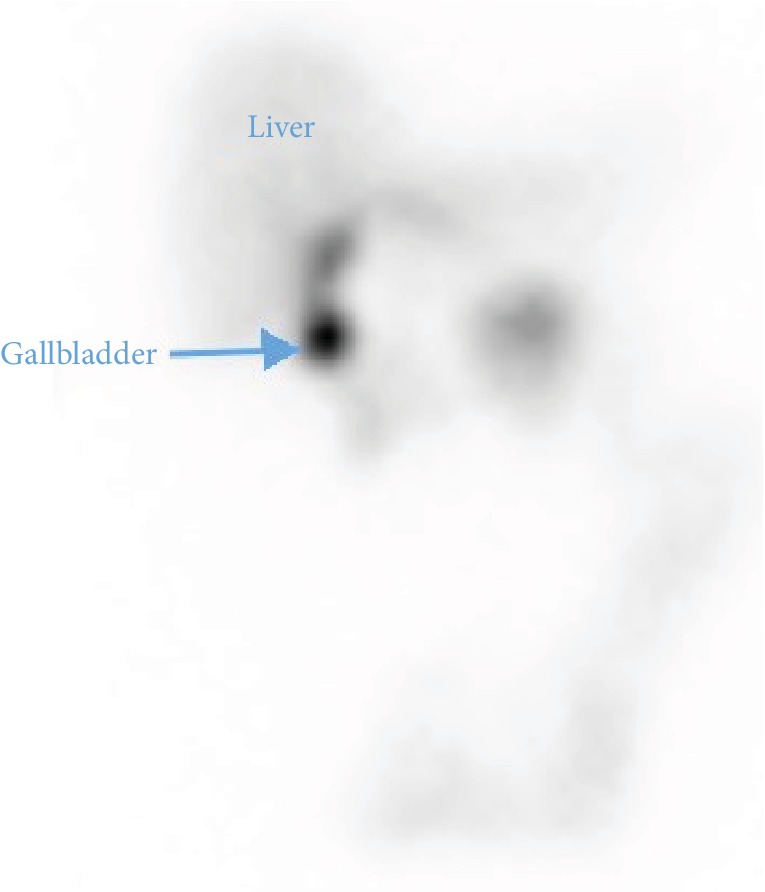
HIDA scan demonstrating early visualization of a small part of the gallbladder.

The next day, the patient developed more pain and defense at the right upper quadrant. Murphy's sign was positive at this time. Temperature was 37.0°C, and heart rate remained around 100 bpm. The HIDA scintigraphy (Figure 2) was performed as well as a dynamic scintigraphy of 20 minutes with a single photon emission computed tomography (SPECT-CT). Adequate hepatic uptake of radionuclide was observed, followed by a rapid biliary tree excretion and visualization of gallbladder activity within 4 minutes. Late acquisitions were not carried out after the appearance of isotopes in the gallbladder. Because of the possibility of a pathologic process other than acute cholecystitis, an abdominal CT was ordered, which was done 24 hours later.

**Figure 3:**
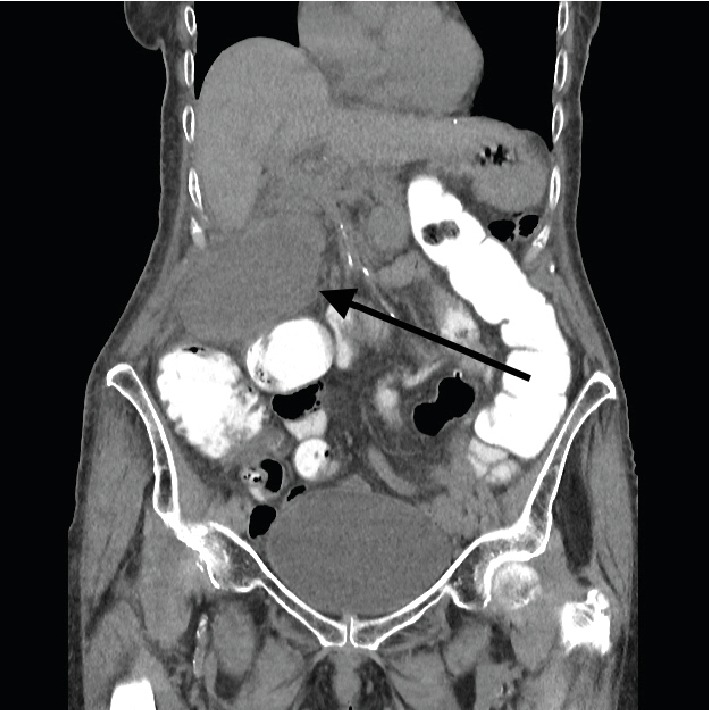
Coronal nonenhanced CT image showing a markedly distended gallbladder (black arrow) and fatty infiltration.

**Figure 4:**
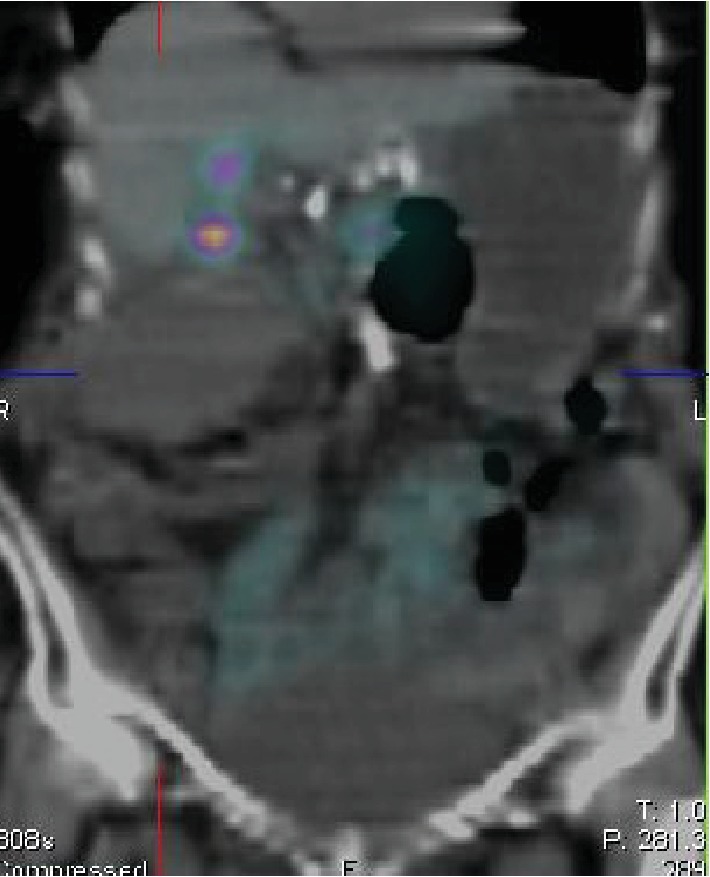
SPECT-CT combining HIDA and CT scans demonstrating discrepancy in gallbladder imaging.

On the noncontrast-enhanced CT, there was dilatation of the gallbladder up to 5 × 5 × 9.5 cm, fatty infiltration surrounding the gallbladder and small amount of free fluid (Figure 3). These findings correlated with the ultrasound but not with the HIDA scintigraphy. By reviewing the HIDA scintigraphy, it became evident that the initially observed gallbladder by HIDA scan was quite small compared to the one seen on the CT. Images were compared with a SPECT-CT (Figure 4). The gallbladder was measured at 9.5 cm on the CT and between 2 and 3 cm on the HIDA scan. The discrepancy in the volume of the gallbladder between these two tests could only be explained by a “missing” part of the gallbladder on the HIDA scintigraphy.

Based on these images showing the blockage of the gallbladder at its midpart without any tumour, a possibility of torsion of the gallbladder was brought up. At this time, the temperature rose to 38.4°C, and the patient had more pain, with defense and rebound tenderness. Facing the ongoing deterioration of the patient, an immediate laparoscopy was planned without undertaking time-consuming additional investigation such as MRI.

During surgery, a volvulus with necrosis of the gallbladder was found and cholecystectomy was carried out. There was a long mesentery and pedicle. The twist was located at a point 2 to 3 cm proximal to the cystic duct. A sharp demarcation between the necrotic and healthy walls of the gallbladder was found. Since the distal part of the gallbladder was healthy with no stone, and since distorted hilar structures could attract the common bile duct, the gallbladder proximal to the neck was divided with an endoscopic stapler leaving a short stump. A drain was left in place. The procedure lasted 30 minutes. Histological examination showed acute necrotic cholecystitis with no stone retrieved.

Postoperative care was uneventful. The patient is still alive one year later.

## 3. Discussion

Torsion of the gallbladder remains an uncommon yet life-threatening situation [8]. It was first reported in 1898 and occurs once in more than 350,000 admissions [1]. Patients are generally over 60, and 79% are females [1] and frequently have associated deformity of the spine [1, 3]. Patients usually present with signs and symptoms suggesting cholecystitis [2, 6–8]. Gallbladder volvulus may also mimic other abdominal pathologies such as tumour of the gallbladder, appendicitis, enterocolitis, and small or large bowel occlusion [9, 10].

The etiology of the torsion always implies an anatomic abnormality that causes the gallbladder to twist [4, 6, 9]. Besides, a torsion-prone mesentery can develop in the elderly with loss of visceral fat, liver atrophy, and visceroptosis with the pedicle attached to the liver [3, 7, 8]. Constitutional anatomic variants are also implicated in the disease [3]. Even though gallstones are present in 32% of the cases, they certainly do not contribute to the condition [1, 6].

Despite the significant improvement in imaging modalities, the diagnosis of volvulus of the gallbladder remains difficult owing to its very low incidence and few specific symptoms. No imaging modality has proven to be sufficiently sensitive to diagnose this condition [2, 4]. Distension of the gallbladder and wall thickening are almost constant, but nonspecific features are seen on both ultrasound and CT scan [1, 2, 5–9]. However, distension of the gallbladder may be massive in volvulus condition compared to acute cholecystitis [2]. Even if the gallstones are detected, impaction of a stone is not present in gallbladder volvulus [2, 8]. Other diagnostic criteria for this condition include a horizontal displaced rather than vertical long axis of the gallbladder, an eccentric location of the gallbladder out of the liver fossa, a lateralized cystic duct to the right of the gallbladder, and fluid between the gallbladder and the gallbladder fossa of the liver [10]. In addition, Doppler ultrasound and contrast-enhanced CT may demonstrate the absence of blood flow in the wall of the gallbladder [5]. Other signs that may suggest a torsion of the gallbladder are the twisted cystic duct seen on ultrasound as a hyperechoic conical structure or as a “knot” [11, 12]. A V-shape distortion of the extrahepatic bile duct and the twisted pedicle may also be seen on multidetector CT [13]. Specific features may also be seen on a CT scan such as “beak and swirl or whirl signs” as they occur in bowel volvulus [11]. HIDA scintigraphy usually demonstrates exclusion of the gallbladder [4].

Diagnosis of torsion relies mainly on the suspicion of the condition. Definitive diagnosis is generally achieved at surgical exploration [2, 8, 12]. Preoperative diagnosis is done in 26% of cases [1] and more frequently in the recent reports [3, 5, 8, 12]. The diagnosis of torsion remains difficult because of its rarity, nonspecific radiologic features [1, 4, 5], and similar presentation to acute cholecystitis [2, 3, 8]. Acalculous cholecystitis, a disease of debilitated patients, does not occur in relatively healthy and stable patients before presentation [1, 7, 9].

Torsion is complete in 82% of cases [1]. Progression to gangrene is very fast and unavoidable [1–6, 10, 13]. Surgery without further delay is mandatory and crucial when the diagnosis is done or suspected [4, 13]. Since the first successful laparoscopic cholecystectomy for an acute case of gallbladder torsion, this procedure has been proven to be a safe and preferred approach [1, 4, 9]. Laparoscopy per se is a valuable diagnostic tool, particularly, in the absence of response to initial treatment or in deteriorating conditions [7]. Drainage of the gallbladder is not a useful course of action, and conservative treatment is not an option [5, 7]. Mortality associated with laparoscopic cholecystectomy is 6% [1].

The patient we present is a typical elderly and skinny female, with a distorted spine. The observed vertebral fractures, on the counterpart, were probably not related to the gallbladder pathology but were surely a red herring. Besides characteristic anatomic features, this case also shares many similarities with other such cases described in the literature: clinical presentation and evolution, radiological imaging with difficult interpretation, delay in diagnosis and intervention, and progression to gallbladder gangrene, but good outcome after surgical treatment.

The possibility of an acute cholecystitis was evoked after the ultrasound showed suggestive signs but no stone. Since gallstones may be missed in acute cholecystitis, a HIDA scintigraphy had been ordered because of the possibility but uncertainty of a diagnosis of acute cholecystitis. Should the HIDA scan demonstrate a complete exclusion of the gallbladder, a diagnosis of acute cholecystitis would have been made, and a conservative treatment could have been “mistakenly” initiated. Antibiotic would not have been useful, and a percutaneous cholecystostomy would have certainly not worked [5].

A part of the gallbladder was evidently seen initially on the HIDA scintigraphy because the torsion was not at the neck. Others reported torsion at a midpoint of the gallbladder [8]. However, such a discrepancy between CT and HIDA using SPECT-CT has not been previously reported. Without reviewing the films, this particular situation would have fallen unrecognized before intervention. Few reports have used HIDA scintigraphy, and in majority of the cases, the gallbladder was completely excluded [4]. This particular case emphasized the necessity for comparing the imaging findings as a crucial step for the diagnosis of torsion [8, 10].

The diagnosis of cholecystitis or volvulus was suspected but not confirmed, even in retrospect, as reported here and in the experience of others [1, 2, 4–9]. Since the patient was clearly deteriorating, the patient was prompted to surgery. Further investigation became unjustifiable as it could have incurred further life-threatening delays. It remained at the time of operation a grade II cholecystitis according to Tokyo guidelines [14]. This case also reiterates the point that laparoscopy must remain a diagnostic tool in difficult cases as well as a treating modality. Laparoscopic cholecystectomy for torsion of the gallbladder in itself is not more challenging than the procedure done for usual acute cholecystitis and carries an excellent prognosis [6, 14].

Considering this case and the review of the literature, we can draw the following conclusions and recommendations:

1. Torsion or volvulus of the gallbladder is a rare but life-threatening condition
2. Preoperative diagnosis is difficult and rarely done even with improvements in diagnostic imaging modalities
3. It should be kept in mind and suspected in elderly and thin female patients with deformity of the spine
4. It should be suspected when ultrasound and/or CT show the following:
  - A distended gallbladder with wall thickening
  - No stone or no impacted stone causing occlusion
  - A horizontal displacement of the long axis of the gallbladder
  - A gallbladder that is outside the liver fossa or “floating”
  - A cystic duct to the right of the gallbladder
5. As the diagnosis is done or suspected, surgical treatment is mandatory and urgent
6. Other modalities (MRI, MRCP, and HIDA) may occasionally be useful but invariably lead to further delay
7. Cholecystectomy is mandatory when facing clinical and radiologic features of severe and deteriorating cholecystitis
8. Laparoscopy may be a useful and necessary diagnostic modality when the condition of patient deteriorates
9. Most cases may be treated with a laparoscopic cholecystectomy, and good outcome is expected after surgery

## 4. Conclusions

Torsion of the gallbladder remains uncommon but its occurrence probably increases as the elderly population grows. Improved imaging techniques allow for more preoperative diagnoses. However, a high index of suspicion remains the mainstay in the diagnostic process. Prompt surgical intervention carries an excellent prognosis.
